# Repeat infusion of autologous bone marrow cells in multiple sclerosis: protocol for a phase I extension study (SIAMMS-II)

**DOI:** 10.1136/bmjopen-2015-009090

**Published:** 2015-09-14

**Authors:** Claire M Rice, David I Marks, Peter Walsh, Nick M Kane, Martin G Guttridge, Juliana Redondo, Pamela Sarkar, Denise Owen, Alastair Wilkins, Neil J Scolding

**Affiliations:** 1 School of Clinical Sciences, University of Bristol, Southmead Hospital, Bristol, UK; 2 Bristol Institute of Clinical Neurosciences, Southmead Hospital, Bristol, UK; 3 Adult BMT Unit, Bristol Royal Hospital for Children, University Hospitals Bristol NHS Foundation Trust & University of Bristol, St Michael's Hill, Bristol, UK; 4 NHS Blood and Transplant, Bristol, UK

**Keywords:** IMMUNOLOGY

## Abstract

**Introduction:**

The ‘Study of Intravenous Autologous Marrow in Multiple Sclerosis (SIAMMS)’ trial was a safety and feasibility study which examined the effect of intravenous infusion of autologous bone marrow without myeloablative therapy. This trial was well tolerated and improvement was noted in the global evoked potential (GEP)—a neurophysiological secondary outcome measure recording speed of conduction in central nervous system pathways. The efficacy of intravenous delivery of autologous marrow in progressive multiple sclerosis (MS) will be examined in the phase II study the ‘Assessment of Bone Marrow-Derived Cellular Therapy in Progressive Multiple Sclerosis (ACTiMuS; NCT01815632)’. In parallel with the ‘ACTiMuS’ study, the current study ‘SIAMMS-II’ will explore the feasibility of repeated, non-myeloablative autologous bone marrow-derived cell therapy in progressive MS. Furthermore, information will be obtained regarding the persistence or otherwise of improvements in conduction in central nervous system pathways observed in the original ‘SIAMMS’ study and whether these can be reproduced or augmented by a second infusion of autologous bone marrow-derived cells.

**Methods and analysis:**

An open, prospective, single-centre phase I extension study. The six patients with progressive MS who participated in the ‘SIAMMS’ study will be invited to undergo repeat bone marrow harvest and receive an intravenous infusion of autologous, unfractionated bone marrow as a day-case procedure. The primary outcome measure is the number of adverse events, and secondary outcome measures will include change in clinical rating scales of disability, GEP and cranial MRI.

**Ethics and dissemination:**

The study has UK National Research Ethics Committee approval (13/SW/0255). Study results will be disseminated via peer-reviewed publications and conference presentations.

**Trial registration number:**

NCT01932593.

**Strengths and limitations of this study**

- Regulated clinical trial of cellullar therapy for progressive multiple sclerosis.
- Extension data for phase I clinical trial.
- Open label trial.
- Small sample size.

## Introduction

Although effective treatments for relapsing–remitting multiple sclerosis (MS) are available, there are no proven therapies available to halt or reverse the progressive phase of the disease which ultimately affects the majority of people with MS. There is preclinical evidence to support a reparative role for bone marrow (BM)-derived cells in demyelinating disease and, following on from this, we have begun to explore the potential of autologous, unselected BM cells for repair in progressive MS. The ‘Study of Intravenous Autologous Marrow in Multiple Sclerosis’ (SIAMMS) was a safety and feasibility study of intravenous autologous BM infusion in patients with progressive MS.^1^ This study was well tolerated and also raised the possibility of partial repair; conduction times in multiple central nervous system (CNS) pathways collated as a composite score (global evoked potential, GEP)^2^ ^3^ improved in all patients studied (n=6).^1^ A randomised, placebo-controlled trial will determine whether autologous BM infusion exerts genuine reparative effects in progressive MS (‘ACTiMuS’; NCT01815632)^4^ but the purpose of ‘SIAMMS-II’ is to explore whether the improvements observed in the initial study performed over 5 years ago have persisted and whether these can be repeated or augmented.

## Methods and design

### Objective and hypothesis

Our hypothesis is that intravenously delivered autologous BM cell therapy in chronic MS has reparative properties. We postulate that BM-derived cells contribute to repair within the CNS via a multiplicity of mechanisms including immunomodulation *and* reparative *and/or* neuroprotective effects. Furthering our understanding of these processes will enable development and refinement of cell therapy for progressive MS.

The phase II ‘ACTiMuS’ trial will explore the efficacy of intravenous infusion of autologous BM-derived cell therapy in progressive MS and its laboratory arm will explore the underlying mechanisms of any observed effect. ‘SIAMMS-II’ will run in parallel with ‘ACTiMuS’ and will investigate whether the previously observed effects can be replicated and/or augmented.

### Trial design

**Figure 1:**
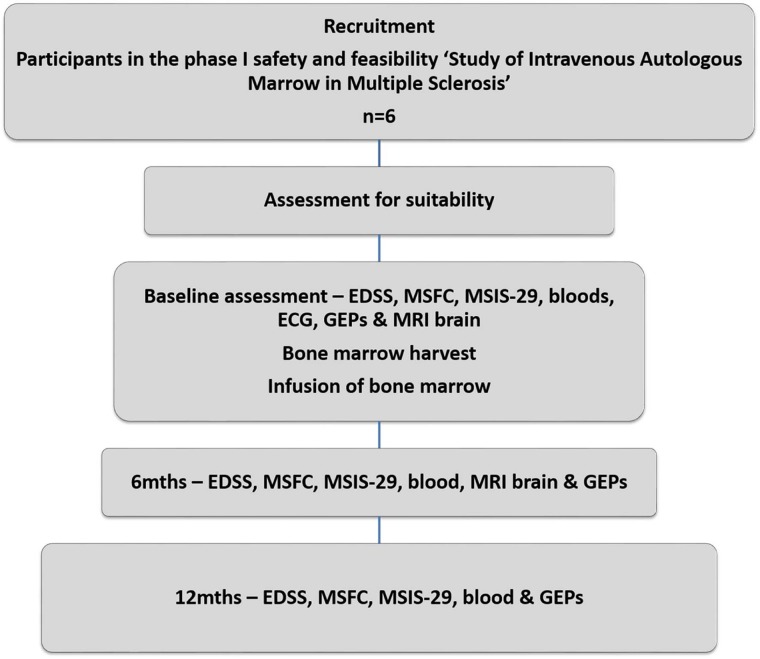
Study schema for the ‘SIAMMS-II’ trial (EDSS, Expanded Disability Status Scale; GEP, global evoked potential; MSFC, Multiple Sclerosis Functional Composite; MSIS, MS Impact Scale; SIAMMS, Study of Intravenous Autologous Marrow in Multiple Sclerosis).

‘SIAMMS-II’ is an open, prospective, single-centre, safety and feasibility extension study. The study schema is presented in figure 1.

### Sample size, eligibility and enrolment

The study is limited to the six people who participated in the original ‘SIAMMS’ study, all of whom are under active follow-up at the Bristol and Avon Multiple Sclerosis (BrAMS) Unit, North Bristol NHS Trust, Bristol, UK. All participants have progressive MS and must fulfil the inclusion and exclusion criteria as detailed in table 1. The prior clinical history of the participants is detailed in the manuscript documenting the results of the original SIAMMS study.^1^ At the time of entry to the ‘SIAMMS’ trial, four participants had had no exposure to disease-modifying treatment. One had received prior treatment with azathioprine and methotrexate and another participant had been previously treated with glatiramer and Avonex. In the intervening period since receiving the first infusion of autologous BM, none of the six participants have received additional disease-modifying therapy.

**Table 1:** Eligibility criteria for the ‘Study of Intravenous Autologous Marrow in Multiple Sclerosis (SIAMMS-II)’ trial

Inclusion criteria	Exclusion criteria
Participation in the phase I safety and feasibility ‘SIAMMS’ (REC reference number 05/Q1704/137)^1^	Pregnancy, breast feeding or lactation History of autologous/allogeneic bone marrow transplantation or peripheral blood stem cell transplant other than in SIAMMS Bone marrow insufficiency History of lymphoproliferative disease or previous total lymphoid irradiation Immune deficiency History of current or recent (<5 years) malignancy Chronic or frequent drug-resistant bacterial infections or presence of active infection requiring antimicrobial treatment Frequent and/or serious viral infection Systemic or invasive fungal disease within 2 years of entry to study Significant renal, hepatic, cardiac or respiratory dysfunction Contraindication to anaesthesia Bleeding or clotting diathesis Current or recent (within preceding 12 months) immunomodulatory therapy other than corticosteroid therapy Treatment with corticosteroids within the preceding 3 months Radiation exposure in the past year other than chest/dental X-rays Previous claustrophobia The presence of any implanted metal or other contraindication to MRI Participation in another experimental study or treatment within previous 24 months


### Trial interventions

Participants will have a BM harvest and reinfusion of autologous marrow as a day-case procedure. A short general anaesthetic will be given for the BM harvest which will be taken from the posterior iliac crests. Approximately 600 mL marrow will be collected together with a single BM trephine. The marrow aspirate will be processed by the National Health Service Blood and Transplant (NHSBT; filtered, bagged and labelled) prior to intravenous infusion.

Assuming specific written informed consent is granted, a BM trephine and a small sample of the BM aspirate will be retained for research. Additional blood samples for research purposes may be requested throughout the duration of the study.

### Outcome measures

#### Primary outcome measure

The primary outcome measure is the number of adverse events (AEs). For the purposes of the study, an AE is defined as any unfavourable and unintended sign, symptom or illness that develops or worsens during the period of the study. This is irrespective of the likelihood that the AE is related to study interventions. AEs may be expected or unexpected and include unwanted side effects, toxicity or sensitivity reactions, as well as abnormal laboratory results, injury or intercurrent illnesses.

A serious adverse event (SAE) is defined as an AE which results in death, is life threatening or requires hospitalisation or prolongation of inpatient stay or which results in persistent or significant disability or incapacity. Any congenital anomaly or birth defect or any event considered to be a medical event of importance will also be classified as a SAE. All SAEs must be reported to the trial coordinating centre as soon as possible. Those hospital admissions that were planned prior to trial entry will not be recorded as SAEs.

As per the ‘ACTiMuS’ trial (personal communication, Rice C, Marks D, Ben-Shlomo Y, *et al*, 2015), expected AEs include:

- Local bruising and discomfort following BM harvest;
- Increase in lower limb spasticity following BM harvest;
- Acute urinary retention following BM harvest;
- Temporary exacerbation of MS following general anaesthesia;
- Hypovolaemia or anaemia following blood and marrow donation;
- Exacerbation of MS due to sepsis, for example, urinary tract infection or chest infection;
- Assessment at or admission to hospital following fall.

Bloods taken for safety analyses will be screened as follows: urea and electrolytes, liver function tests, full blood count with differential white cell count, coagulation, group and save, C reactive protein, glucose, calcium, magnesium, chloride, bicarbonate, phosphate, viral serology (including cytomegalovirus, Epstein-Barr virus, herpes simplex virus, varicella zoster virus, toxoplasmosis, hepatitis B and C, HIV, human T-cell lymphoma virus and syphilis screening. Urinalysis (microscopy and culture) will also be performed.

#### Secondary outcome measures

Change in clinical measures of disability, GEP and cranial MRI findings are included as secondary outcome measures.

Clinical outcomes will be assessed at entry and at 6 months and 1 year. The clinical rating scales will include the widely used Expanded Disability Status Scale (EDSS)^5^ together with the Multiple Sclerosis Functional Composite (MSFC).^6^ The latter is a three-part quantitative assessment including a timed walk, nine-hole peg test and Paced Auditory Serial Addition Test (PASAT). In addition, participants will be asked to complete the MS Impact Scale (MSIS-29) which is a well-validated patient-completed rating scale.^7–10^ The measures taken for clinical secondary outcome measures will be:

1. Physician-based EDSS: time to EDSS progression of at least one point from a baseline EDSS of 4.0, 4.5 or 5.0 or at least 0.5 points from a baseline EDSS ≥5.5;
2. Patient-based MSIS-29 physical impact scale V.2: overall mean change from baseline to end of study;
3. MSFC: overall mean change of z-scores, from baseline to final visit.

Multimodal evoked potentials will be examined at 0, 6 and 12 months. Evoked potential abnormalities will be quantified according to a four-point graded ordinal score modified from Leocani *et al*^3^ (0=normal; 1=increased latency; 2=increased latency and abnormal amplitude; 3=absent) and the composite GEP score calculated.

The recording of the evoked potentials shall be in accordance with the Guidelines of the International Federation of Clinical Neurophysiology^11^ and analysis will be performed using standard methods^12^ (box 1). Electrophysiological responses shall be considered abnormal if they exceed 2.5 SDs of the normal values or cannot be detected.

Box 1 **Method for recording of multimodal evoked potentials**

Visual evoked potentials (VEPs) will be evoked with a rear-projected chequer board pattern using an optomechanical device subtending 30° at the retina, check-size 1°, white brightness of 150/cdm^2^ and contrast 87.5%.

Monaural stimulation will be delivered via earphones to each side with rarefaction click stimuli of 0.1 ms duration at an intensity of 75 dB above the subjective hearing threshold while the contralateral ear was masked with white noise.

Sensory evoked potentials (SEPs) will be obtained by delivering electrical stimulation with square wave pulses of 0.2 ms duration to the median and the posterior tibial nerves, at the wrist and ankle, respectively.

Motor evoked potentials (MEPs) will be recorded from electrodes situated over the abductor pollicis brevis muscle in the hand and the abductor hallucis in the foot using a 9 cm circular coil held over the vertex. The central motor conduction time (CMCT) was calculated by subtracting ½(M+F+1) from the MEP latency where M is the distal motor latency and F is the minimum F wave latency.

The global evoked potential score will then be calculated as the sum of left and right brainstem auditory evoked potential and VEP scores (0–12) and left and right upper and lower SEPs (0–12) and CMCTs (0–12).

Participants will undergo cranial MRI at entry and at 6 months after BM infusion. The secondary MRI outcome measures will relate to lesion load and atrophy measures of the brain.

Annual subjective patient and treating physician assessments of efficacy will also be recorded.

### Trial status

‘SIAMMS-II’ opened to recruitment in March 2014 and is ongoing.

### Analysis

A full statistical analysis plan will be written prior to data collection. The null hypothesis is that there will be no significant difference in the primary and secondary outcomes between intervention and control arms at 12 months.

Secondary outcomes will be scored according to standard methodology, but the limitations of the small sample size are acknowledged.

### Conclusion

On the background of extensive preclinical studies and anticipated low risk of significant harm, we started a phase I trial intravenous delivery of filtered but otherwise unmodified autologous BM in 2006. The successful completion of this early trial and the suggestion that electrophysiological improvement may have occurred,^1^ made further exploration of the reparative potential of autologous marrow in MS mandatory. We have begun to assess the efficacy of this approach in the randomised, double-blind ‘ACTiMuS’ trial (personal communication, Rice C, Marks D, Ben-Shlomo Y, *et al*, 2015). However, ‘SIAMMS-II’ will give some preliminary information about the value of retreating progressive MS with repeat infusion of autologous BM.

There is now a wealth of preclinical data which supports a clear scientific rationale for BM-derived cell therapy in MS. This, together with the extensive clinical experience of BM transplantation which has been acquired over several decades, justifies the examination of the putative clinical benefit of BM-derived cell therapy for MS in clinical trials. Indeed, in addition to our own studies using filtered but otherwise unselected BM, a number of clinical trials are now exploring the safety and therapeutic effectiveness of BM-derived cell therapy for MS using specific subpopulations of BM cells. We and others have recently reviewed the approaches being explored^13–15^ but, while candidates certainly include multipotent mesenchymal stromal cells, the cell population(s) of greatest therapeutic potential have not been definitively identified. The rationale for our use of unfractionated marrow has been set out in detail elsewhere,^1^ but in essence our approach utilises the potential reparative effects of multiple cell populations and has not be shown to be associated with increased clinical risk.

‘SIAMMS-II’, the ‘ACTiMuS’ trial and other ongoing studies will determine whether BM-derived cell therapy genuinely effects neurological repair in MS and will further understanding of the potential multiplicity of reparative mechanisms. Optimisation of treatment is likely to be an iterative process dependent on efficient back-translation of information gained from carefully designed clinical trials, but it is hoped that future refinements will exploit more efficiently the therapeutic potential of BM cell therapy for the treatment of progressive MS.

## References

[1] Rice CM, Mallam EA, Whone AL Safety and feasibility of autologous bone marrow cellular therapy in relapsing-progressive multiple sclerosis. Clin Pharmacol Ther 2010;87:679–85. 10.1038/clpt.2010.44 20445531

[2] Leocani L, Medaglini S, Comi G Evoked potentials in monitoring multiple sclerosis. Neurol Sci 2000;21 (4 Suppl 2):S889–91. 11205369 10.1007/s100720070032

[3] Leocani L, Rovaris M, Boneschi FM Multimodal evoked potentials to assess the evolution of multiple sclerosis: a longitudinal study. J Neurol Neurosurg Psychiatry 2006;77:1030–5. 10.1136/jnnp.2005.086280 16735397 PMC2077734

[4] Rice CM, Marks DI, Ben-Shlomo Y Assessment of bone marrow-derived cellular therapy in progressive multiple sclerosis (ACTiMuS): study protocol for a randomised, placebo-controlled, stepped wedge study. Trials 2015. In press. 10.1186/s13063-015-0953-1 PMC4606493 26467901

[5] Kurtzke JF Rating neurologic impairment in multiple sclerosis: an expanded disability status scale (EDSS). Neurology 1983;33: 1444–52. 10.1212/WNL.33.11.1444 6685237

[6] Fischer JS, Rudick RA, Cutter GR The Multiple Sclerosis Functional Composite Measure (MSFC): an integrated approach to MS clinical outcome assessment. National MS Society Clinical Outcomes Assessment Task Force. Mult Scler 1999;5:244–50. 10.1177/135245859900500409 10467383

[7] Costelloe L, O'Rourke K, McGuigan C The longitudinal relationship between the patient-reported Multiple Sclerosis Impact Scale and the clinician-assessed Multiple Sclerosis Functional Composite. Mult Scler 2008;14:255–8. 10.1177/1352458507081274 17942522

[8] Hobart J, Lamping D, Fitzpatrick R The Multiple Sclerosis Impact Scale (MSIS-29): a new patient-based outcome measure. Brain 2001;124 (Pt 5):962–73. 10.1093/brain/124.5.962 11335698

[9] Hobart JC, Riazi A, Lamping DL How responsive is the Multiple Sclerosis Impact Scale (MSIS-29)? A comparison with some other self report scales. J Neurol Neurosurg Psychiatry 2005;76:1539–43. 10.1136/jnnp.2005.064584 16227547 PMC1739386

[10] Hoogervorst EL, Zwemmer JN, Jelles B Multiple Sclerosis Impact Scale (MSIS-29): relation to established measures of impairment and disability. Mult Scler 2004;10:569–74. 10.1191/1352458504ms1078oa 15471375

[11] Deuschl G, Andrew E Recommendations for the practice of clinical neurophysiology: guidelines of the International Federation of Clinical Neurophysiology. Amsterdam, NY: Elsevier, 1999. 10617380

[12] Walsh P, Kane N, Butler S The clinical role of evoked potentials. J Neurol Neurosurg Psychiatry 2005;76 (Suppl 2):ii16–22. 10.1136/jnnp.2005.068130 15961863 PMC1765695

[13] Rice CM, Kemp K, Wilkins A Cell therapy for multiple sclerosis: an evolving concept with implications for other neurodegenerative diseases. Lancet 2013;382:1204–13. 10.1016/S0140-6736(13)61810-3 24095194

[14] Xiao J, Yang R, Biswas S Mesenchymal stem cells and induced pluripotent stem cells as therapies for multiple sclerosis. Int J Mol Sci 2015;16:9283–302. 10.3390/ijms16059283 25918935 PMC4463588

[15] Holloman JP, Ho CC, Hukki A The development of hematopoietic and mesenchymal stem cell transplantation as an effective treatment for multiple sclerosis. Am J Stem Cells 2013;2:95–107. 23862098 PMC3708509

